# Sarcopenia as a prognostic indicator in colorectal cancer: an updated meta-analysis

**DOI:** 10.3389/fonc.2023.1247341

**Published:** 2023-10-27

**Authors:** Jie He, Wei Luo, Yuanyuan Huang, Lingmeng Song, Yang Mei

**Affiliations:** ^1^ Clinical Medical College of Chengdu Medical College, Chengdu, Sichuan, China; ^2^ Department of Pulmonary and Critical Care Medicine, The First Affiliated Hospital of Chengdu Medical College, Chengdu, Sichuan, China; ^3^ Key Laboratory of Geriatric Respiratory Diseases of Sichuan Higher Education Institutes, Chengdu, Sichuan, China; ^4^ Radiology Department, The First Affiliated Hospital of Chengdu Medical College, Chengdu, Sichuan, China; ^5^ Medical Department, The First Affiliated Hospital of Chengdu Medical College, Chengdu, Sichuan, China

**Keywords:** sarcopenia, skeletal muscle mass, colorectal cancer, prognosis, meta-analysis

## Abstract

**Background:**

Sarcopenia, often observed in the elderly, is associated with declining skeletal muscle mass and impaired muscle function. This condition has been consistently linked to a less favorable prognosis in various malignancies. Computed tomography (CT) is a frequently employed modality for evaluating skeletal muscle mass, enabling the measurement of the skeletal muscle index (SMI) at the third lumbar vertebra (L3) level. This measurement serves as a defining criterion for sarcopenia. The meta-analysis dealt with evaluating the promise sarcopenia held as a prognostic indicator in individuals with colorectal cancer.

**Methods:**

Research relevant to the subject was determined by systematically searching PubMed, Embase, Web of Science, WANFANG, and CNKI (up to June 11, 2023, published studies). In this meta-analysis, the incidence of sarcopenia in individuals with colorectal cancer was combined to analyze the disease-free survival (DFS), overall survival (OS), and cancer-specific survival (CSS) of these individuals with and without sarcopenia. The included research was evaluated for quality per the Newcastle-Ottawa Scale (NOS) score. In the multivariate analysis of each study, the direct extraction of hazard ratio (HR) with a 95% confidence interval (CI) was executed. STATA 11.0 was applied to integrate and statistically analyze the data.

**Results:**

Overall 20 articles participated in this meta-analysis. A 34% incidence of sarcopenia was noted in colorectal cancer. The presence of sarcopenia denoted a decrease in OS (HR=1.72,95% CI=1.45-2.03), DFS (HR=1.42,95% CI=1.26-1.60) and CSS (HR=1.48,95% CI=1.26-1.75) in individuals with colorectal cancer. In addition, the subgroup analysis depicted a pattern consistent with the overall analysis results.

**Conclusion:**

CT-defined sarcopenia exhibits promise as an indicator of survival prognosis in individuals with colorectal cancer. Future studies need a more rigorous definition of sarcopenia to further verify these findings.

**Systematic review registration:**

https://www.crd.york.ac.uk/PROSPERO/, identifier CRD42023431435.

## 1 Introduction

Globally, Colorectal cancer is the third highly predominant malignancy and stands as the fourth highest contributor to cancer death in men and women, with rectal cancer accounting for approximately 30% of these cases (1, 2). Colorectal cancer has been challenging to address due to its anatomical structure and high local recurrence rate. Currently, the standard treatment encompasses surgical resection and neoadjuvant chemoradiotherapy (nCRT) for individuals with locally advanced rectal cancer (3). Preoperative nCRT has been noted to improve local infiltration and reduce toxicity in rectal cancer compared with postoperative nCRT, with no improvement in overall survival (OS) (4). Although the treatment of colon cancer has progressed greatly, including surgery or radiotherapy, the prognosis of individuals with colon cancer have not progressed significantly in recent years (5). Prognostic stratification of colorectal cancer patients relies on tumor pathology after radical surgery; however, baseline host-related factors may also negatively impact long-term survival outcomes. Therefore, identifying key risk factors linked with the prognosis of individuals with colorectal cancer patients is of great clinical value.

It is well known that the body structure of cancer patients changes as the disease progresses (6). The European Working Group on Sarcopenia in the Elderly (EWGSOP), associate sarcopenia with declining muscle mass and impairment of muscle function (strength or performance) (7). A frequently observed condition in older adults, sarcopenia has been consistently linked with a more unfavorable prognosis across diverse cancers, including head and neck, colorectal, and breast cancers (8–12). Meyer et al. found that sarcopenia was a prognostic marker for survival in gastric cancer undergoing palliative chemotherapy (13). Additionally, Gan H et al. pointed out both low skeletal muscle mass and poor muscle quality were relative with poor long term survival of patients with pancreatic cancer (14). The Computed tomography (CT) scans enable the measurement of the third lumbar spine skeletal muscle index (L3-SMI). This measurement serves as a defining criterion for sarcopenia (15). In individuals with cancer, routine CT scans are utilized for assessing tumor lesions and monitoring tumor metastases. The scan data is commonly utilized for measuring skeletal muscle mass without additional radiation.

The relationship of sarcopenia with cancer outcomes is garnering growing attention. The data of prior meta-analyses is indicative of the reduced efficacy of sarcopenia in predicting OS in colorectal cancer patients (16, 17). Several more recent studies have reported a correlation of sarcopenia with OS in individuals with colorectal cancer, however, the results remain debatable. Herein, a meta-analysis investigating the potential negative impact of sarcopenia, as defined by L3-SMI, on OS in individuals with colorectal cancer was conducted.

## 2 Methods

This meta-analysis study adhered to the guidelines provided in the Preferred Reporting Items for Systematic Evaluation and Meta-Analysis (PRISMA) (18).

### 2.1 Search strategy

Two researchers undertook the task of assessing the literature pool for relevant research independently. In case of disagreements, a third researcher was involved in resolving the issues through discussion, if required. A pre-defined protocol was utilized to conduct the research. The literature relevant to the research was filtered by assessing published articles (up to June 1, 2023) available on PubMed, Embase, Web of Science, WANFANG, and CNKI. The search was focused on the key terms and limited to the English language. The below-mentioned MeSH/main keywords were utilized: “colorectal”, “rectal”, “rectum”, “colon”, “colonic”, “myopenia”, “sarcopenia”, and “muscle mass” using datasets. In addition, studies and relevant reviews were manually searched to determine additional eligible studies.

### 2.2 Inclusion and exclusion criteria

Studies were part of this meta-analysis if they met the mentioned criteria: (1) individuals with colorectal cancer; (2) comparing OS, disease-free survival (DFS), and cancer-specific survival (CSS) in patients with and without sarcopenia; (3) quantitative measurement of skeletal muscle mass by CT at the L3 level; (4) studies reporting adjusted hazard ratios (HR) and 95% confidence intervals (CI) for OS, DFS, and CSS studies.

The exclusion of case reports, reviews, conference abstracts, commentaries, and animal studies were carried out. In addition, research that lacked valid data was excluded, such as HR and 95% CI.

### 2.3 Data extraction

Standardized forms were utilized by two researchers for pertinent data extraction from the included studies. A third researcher was involved in the resolution of disagreements through consensus and discussion. Each study involved in the research was assessed for the following data: first author, publication year, country, design of the study design, patient number, mean/median population age, percentage of males, stage of the disease, SMI measure, the cut-off point for SMI, the prevalence of sarcopenia, and HR for OS, DFS, and 95% CI for CSS. OS was termed as the occurrence of fatality due to any cause; DFS as the recurrence of the disease or fatality; and CSS as the occurrence of fatality linked with cancer.

### 2.4 Quality assessment

The Newcastle-Ottawa Scale (NOS) assessed the quality of the research involved in the study. This assessment was conducted by two independent reviewers, with the quality ranging from low to medium to high per respective scores of 1-3, 4-6, and 7-9 (19). The two reviewers engaged in discussion for the resolution of any discrepancy in the above studies.

### 2.5 Statistical analysis

This meta-analysis was executed through STATA 11.0. Direct extraction of sarcopenia-linked 95% CIs for OS, DFS, and CSS, as well as HRs was carried out utilizing the included studies. Chi-square tests and *I^2^* statistics were employed for assessing heterogeneity across the studies with *P* < 0.1 and *I^2^* > 50% suggesting statistical significance. Given the variation in cut-off points for the diagnosis of sarcopenia in SMI, some heterogeneity may exist between studies. Therefore, the random-effects model was utilized for multivariate data when *P* < 0.1 and *I^2^* > 50% in this research; conversely, the fixed-effect model was employed. For combined studies with the number of studies greater than 5, a subgroup analysis was performed. Furthermore, the heterogeneity sources were explored through sensitivity analyses. Whereas possible potential bias was examined through funnel plots with low bias indicated by the symmetrical distribution of funnel plot shapes. *P* < 0.05 from Begg’s and Egger’s tests was considered publication bias for the combined studies.

## 3 Results

### 3.1 Characteristics of the literature

**Figure 1:**
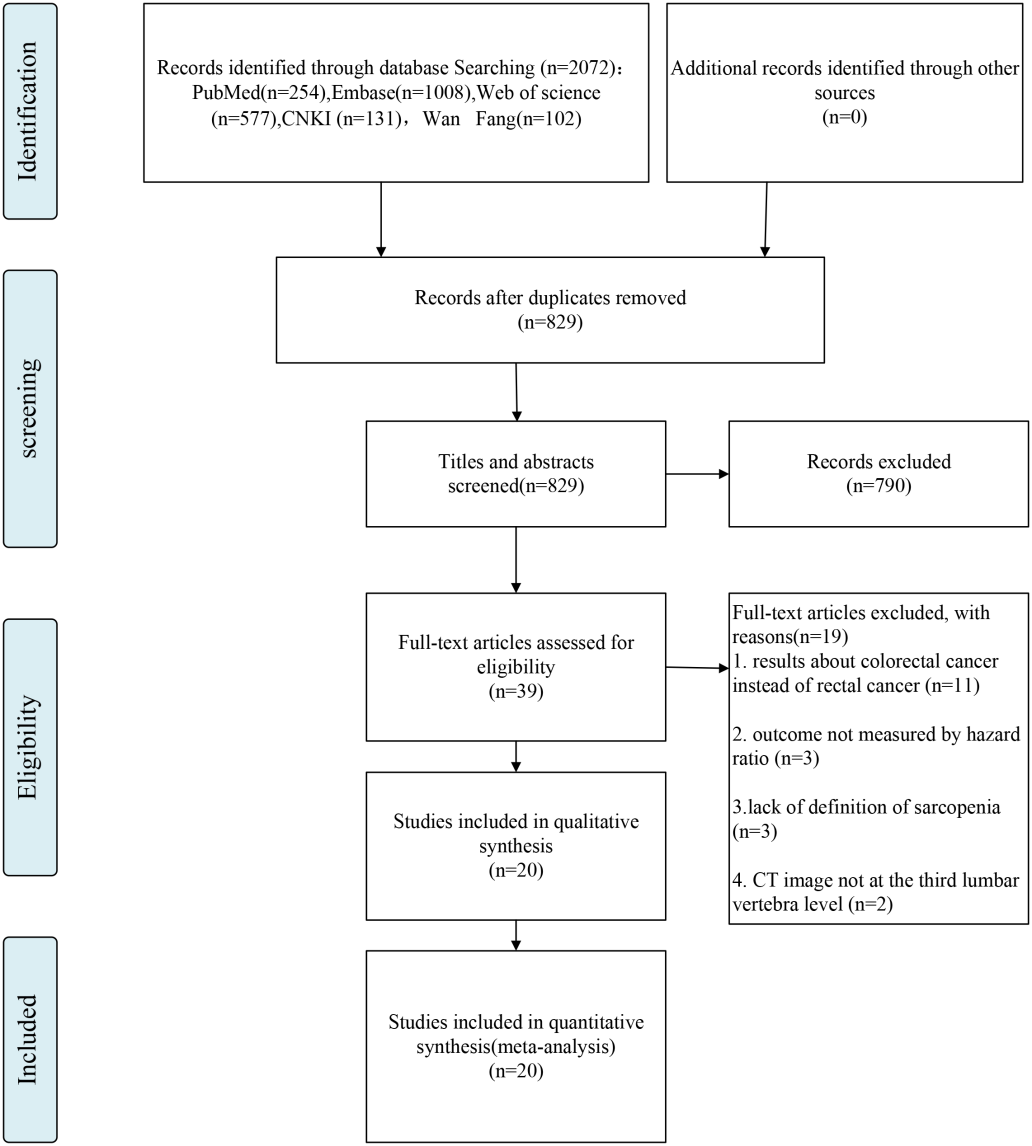
Flow chart of inclusion criteria in the study.

Overall, 829 published studies were determined according to our search, and duplicate publications were eliminated. Further, the titles, abstracts, and even the full articles were read, and finally, 20 articles were involved in this meta-analysis (12, 20–38). The search strategy is depicted in a flowchart in **Figure 1**, which covers the reasons for the exclusion criterion. Among the included studies, two study were prospective and the other 18 were retrospective studies. In addition, 13 studies were conducted in an Asian country and the other studies were from European and North American countries. Moreover, 15 study had an NOS score of >5. **Table 1** depicts the summarized data concerning the features of the included studies. Furthermore, **Table 2** summarizes the definition and critical values of sarcopenia in the assessed studies.

**Table 1:** Characteristics of included studies.

Author	Year	Study type	Country	Groups	Number	Age(years)	Male/female (%)	BMI (kg/m2)	Prevalence of sarcopenia (%)	tumor type	Stage	NOS
Choi et al. (23)	2018	Retrospective	South Korea	Sarcopenia	74	Mean 64.2	61/13	Mean 22.4	39.4	rectal cancer	II-III	5
				Non-sarcopenia	114	Mean 59.5	56/58	Mean 24.7			II-III	
Malietzis et al. (21)	2016	Prospective	UK	Sarcopenia	485	Median 69	NA	NA	NA	colorectal cancer	I-IV	6
				Non-sarcopenia	320	NA	NA	NA			I-IV	
Nakanishi et al. (37)	2018	Retrospective	Japan	Sarcopenia	298	Mean66.5	188/110	Mean21.0	NA	colorectal cancer	I-IV	6
				Non-sarcopenia	196	Mean65.4	110/86	Mean24.1			I-IV	
Miyamoto et al. (20)	2015	Retrospective	Japan	Sarcopenia	55	Median 74.0	34/21	Median 21.0	NA	colorectal cancer	I-III	6
				Non-sarcopenia	165	Median 68.0	101/64	Median 23.3			I-III	
Sueda et al. (25)	2018	Retrospective	Japan	Sarcopenia	105	NA	41/64	NA	NA	colorectal cancer	I-III	6
				Non-sarcopenia	106	NA	93/13	NA			I-III	
Feliciano et al. (22)	2017	Retrospective	USA	Sarcopenia	1133	NA	NA	NA	NA	colorectal cancer	I-III	5
				Non-sarcopenia	1337	NA	NA	NA			I-III	
Park et al. (24)	2018	Retrospective	South Korea	Sarcopenia	40	NA	NA	NA	38.4	rectal cancer	III-IV	5
				Non-sarcopenia	64	NA	NA	NA			III-IV	
Takeda et al. (26)	2018	Retrospective	Japan	Sarcopenia	37	Mean65.0	26/11	Mean 20.6	23.6	rectal cancer	I-III	6
				Non-sarcopenia	107	Mean60.0	76/31	Mean 23.9			I-III	
Chung et al. (29)	2019	Retrospective	South Korea	Sarcopenia	51	NA	38/13	NA	NA	rectal cancer	I-III	7
				Non-sarcopenia	42	NA	22/20	NA			I-III	
Hopkins et al. (27)	2019	Retrospective	Canada	Sarcopenia	266	NA	170/96	NA	50.4	colorectal cancer	I-III	5
				Non-sarcopenia	702	NA	419/283	NA			I-III	
Bingmer et al. (28)	2020	Retrospective	USA	Sarcopenia	16	Mean62.8	9/7	Mean 22.2	25	rectal cancer	I-IV	5
				Non-sarcopenia	48	Mean59.2	15/33	Mean 28.6			I-IV	
Abe et al. (30)	2022	Retrospective	Japan	Sarcopenia	55	Mean66.0	37/18	Mean 22.1	30.7	rectal cancer	I-IV	8
				Non-sarcopenia	179	Mean64.0	114/65	Mean 22.9			I-IV	
Horie et al. (31)	2022	Retrospective	Japan	Sarcopenia	22	Mean64.5	13/9	Mean 21.8	NA	rectal cancer	I-III	6
				Non-sarcopenia	24	Mean66.8	14/10	Mean 24.9			I-III	
Lee et al. (32)	2021	Retrospective	Korea	Sarcopenia	1155	NA	NA	NA	NA	colorectal cancer	I-III	7
				Non-sarcopenia	1178	NA	NA	NA			I-III	
Abe et al. (33)	2023	Retrospective	Japan	Sarcopenia	306	Mean67.0	221/85	Mean 21.3	NA	rectal cancer	I-IV	8
				Non-sarcopenia	402	Mean62.0	202/200	Mean 22.9			I-IV	
Giani et al. (34)	2022	Prospective	Italy	Sarcopenia	34	NA	NA	NA	26.8	rectal cancer	I-IV	7
				Non-sarcopenia	93	NA	NA	NA			I-IV	
Portale et al. (35)	2023	Retrospective	Italy	Sarcopenia	30	Mean76.0	19/11	Mean 24.7	18.4	rectal cancer	I-III	7
				Non-sarcopenia	135	Mean68.0	95/40	Mean 25.7			I-III	
Gartrell et al. (12)	2023	Retrospective	Australia	Sarcopenia	44	NA	31/13	NA	33.3	rectal cancer	II-III	7
				Non-sarcopenia	88	NA	62/26	NA			II-III	
Xie et al. (36)	2023	Retrospective	China	Sarcopenia	271	Mean64.6	162/109	Mean 18.0	18.8	colorectal cancer	I-IV	8
				Non-sarcopenia	1170	Mean56.6	742/428	Mean 22.8			I-IV	
Han et al. (38)	2020	Retrospective	Korea	Sarcopenia	944	NA	641/303	NA	68.2	rectal cancer	0-III	6
				Non-sarcopenia	440	NA	247/193	NA			0-III	

BMI, body mass index; NOS, Newcastle-Ottawa Scale; NA, not applicable.

**Table 2:** Definition and cutoff values of sarcopenia measured by the third lumbar vertebra skeletal muscle index (L3SMI) in our included studies.

Author	Year	Definition and cutoff values of sarcopenia
Choi et al. (23)	2018	Male:<52.4 cm^2^/m^2^, female:<38.5 cm^2^/m^2^
Malietzis et al. (21)	2016	Male:<52.4 cm^2^/m^2^, female:<38.5 cm^2^/m^2^
Nakanishi et al. (37)	2018	Male:<52.4 cm^2^/m^2^, female:<38.5 cm^2^/m^2^
Miyamoto et al. (20)	2015	Male:<52.4 cm^2^/m^2^, female:<38.5 cm^2^/m^2^
Sueda et al. (25)	2018	Male: if BMI<25kg/m^2^:<43cm2/m^2^; if BMI>25kg/m^2^:<53cm^2^/m^2^, Female:<41 cm^2^/m^2^
Feliciano et al. (22)	2017	For BMI<30kg/m^2^: male:<52cm^2^/m^2^, female:<38cm^2^/m^2^; For BMI>25kg/m^2^: male<54cm^2^/m^2^, female:47cm^2^/m^2^
Park et al. (24)	2018	Male:<52.4 cm^2^/m^2^, female:<38.5 cm^2^/m^2^
Takeda et al. (26)	2018	Male:<45.0 cm^2^/m^2^, female:<33.8 cm^2^/m^2^
Chung et al. (29)	2019	Male:<52.4 cm^2^/m^2^, female:<38.5 cm^2^/m^2^
Hopkins et al. (27)	2019	Male: if BMI<25kg/m^2^:<43cm2/m^2^; if BMI>25kg/m^2^:<53cm2/m2, Female:<41 cm^2^/m^2^
Bingmer et al. (28)	2020	Male:<52.4 cm^2^/m^2^, female:<38.5 cm^2^/m^2^
Abe et al. (30)	2022	PMI: male<6.36 cm^2^/m^2^, female<3.92 cm^2/^m^2^
Horie et al. (31)	2022	PV: male:<140.93 cm^3^/m^2^, female<105.8 cm^3^/m^2^
Lee et al. (32)	2021	Male:<52.4 cm^2^/m^2^, female:<38.5 cm^2^/m^2^
Abe et al. (33)	2023	SMI: male:<52.4 cm^2^/m^2^, female:<38.5 cm^2^/m^2^; PMI: male<6.36 cm^2^/m^2^, female<3.92 cm^2/^m^2^
Giani et al. (34)	2022	Male:<52.4 cm^2^/m^2^, female:<38.5 cm^2^/m^2^
Portale et al. (35)	2023	Male:<52.4 cm^2^/m^2^, female:<38.5 cm^2^/m^2^
Gartrell et al. (12)	2023	Male:<47.5 cm^2^/m^2^, female:<39.1 cm^2^/m^2^
Xie et al. (36)	2023	Man< 6.92 Kg/m^2^, woman< 5.13 Kg/m^2^
Han et al. (38)	2020	Male:<52.4 cm^2^/m^2^, female:<38.5 cm^2^/m^2^

BMI, body mass index; PMI, psoas muscle mass index; PV, psoas muscle volume.

### 3.2 Prevalence of sarcopenia in patients with colorectal cancer

**Figure 2:**
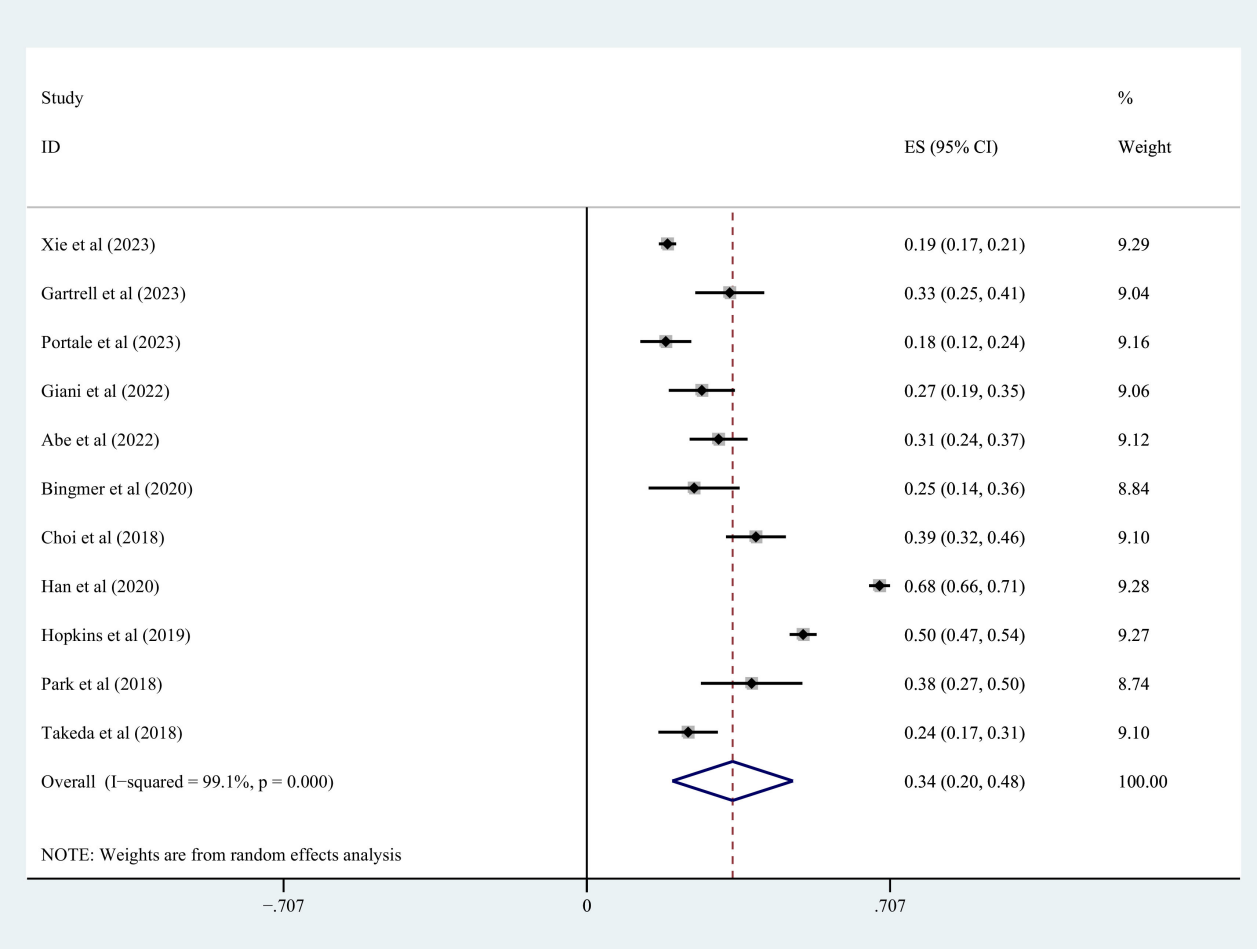
Incidence of sarcopenia in colorectal patients.

The meta-analysis of sarcopenia prevalence in colorectal cancer patients included 11 studies (**Table 1**), involving 4855 individuals overall, with sarcopenia present in 2018 individuals and absent in 2837. In this meta-analysis, the overall prevalence of sarcopenia was calculated in individuals with colorectal cancer to be 34% (95% CI=0.20-0.48, *I^2 = ^* 99.1%, *P*<0.001) (**Figure 2**).

### 3.3 Overall analysis of survival outcomes

**Figure 3:**
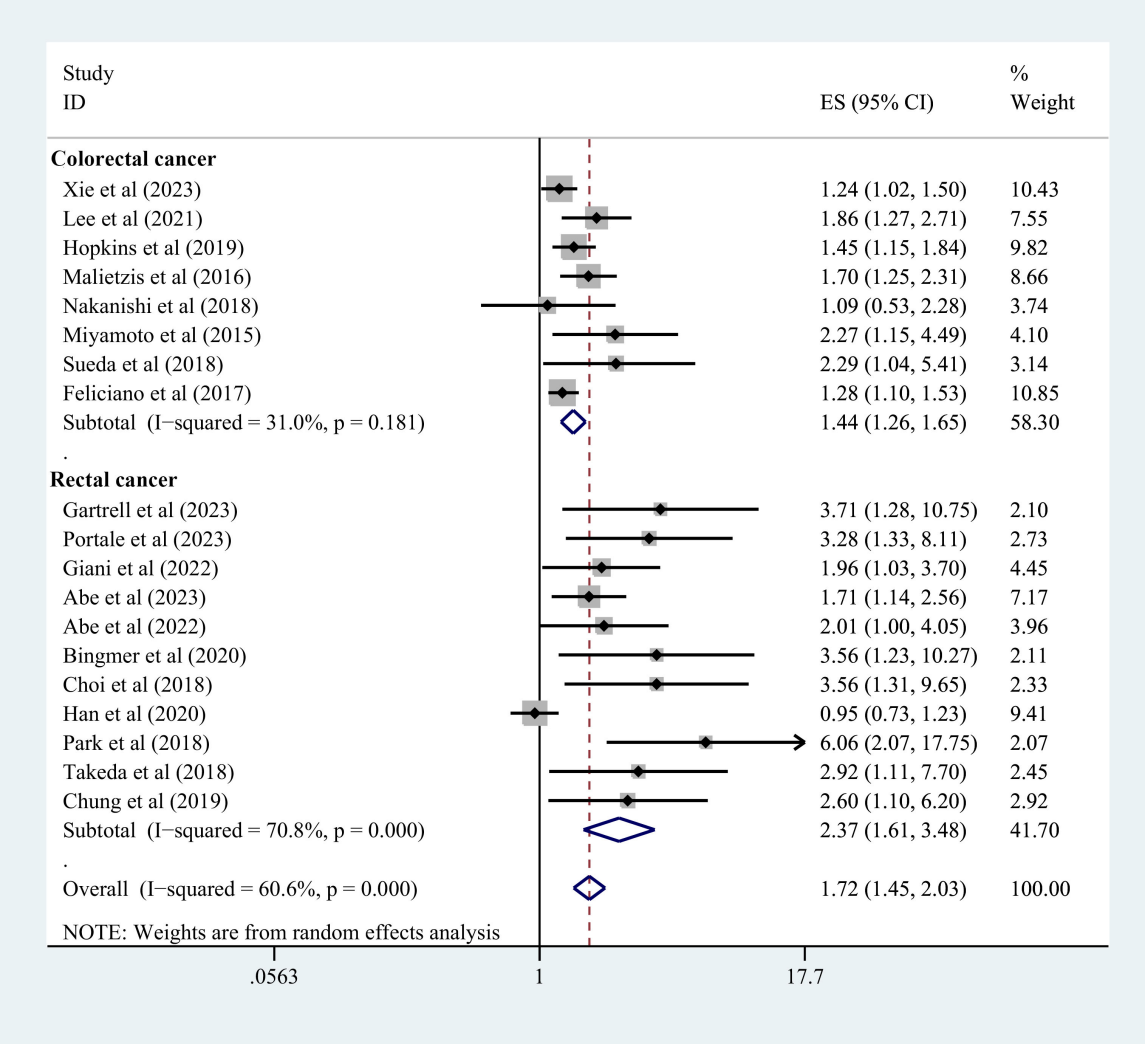
Forest plot of OS risk in colorectal cancer patients with and without sarcopenia. OS, overall survival.

**Figure 4:**
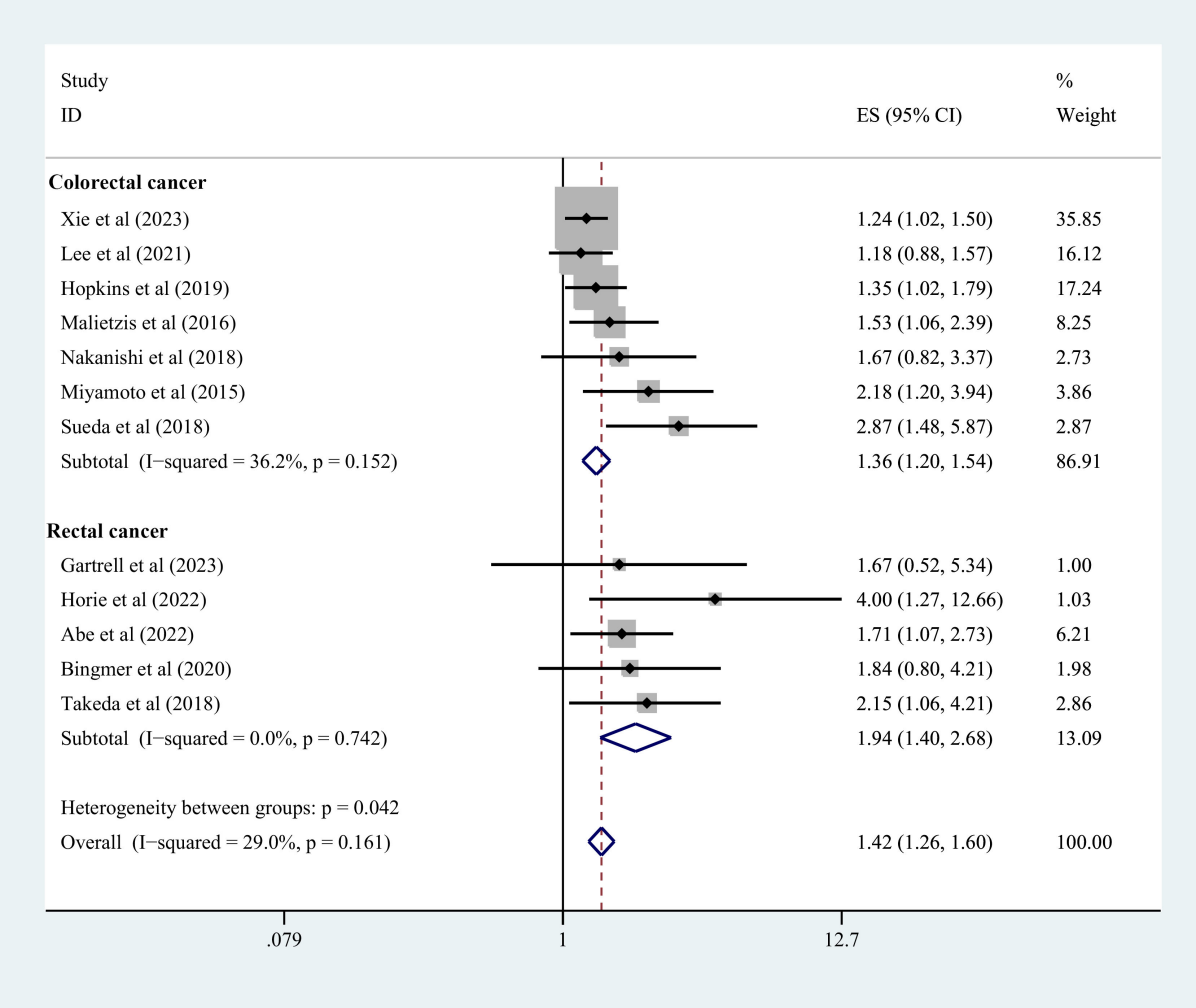
Forest plot of DFS risk in colorectal cancer patients with and without sarcopenia. DFS, disease-free survival.

**Figure 5:**
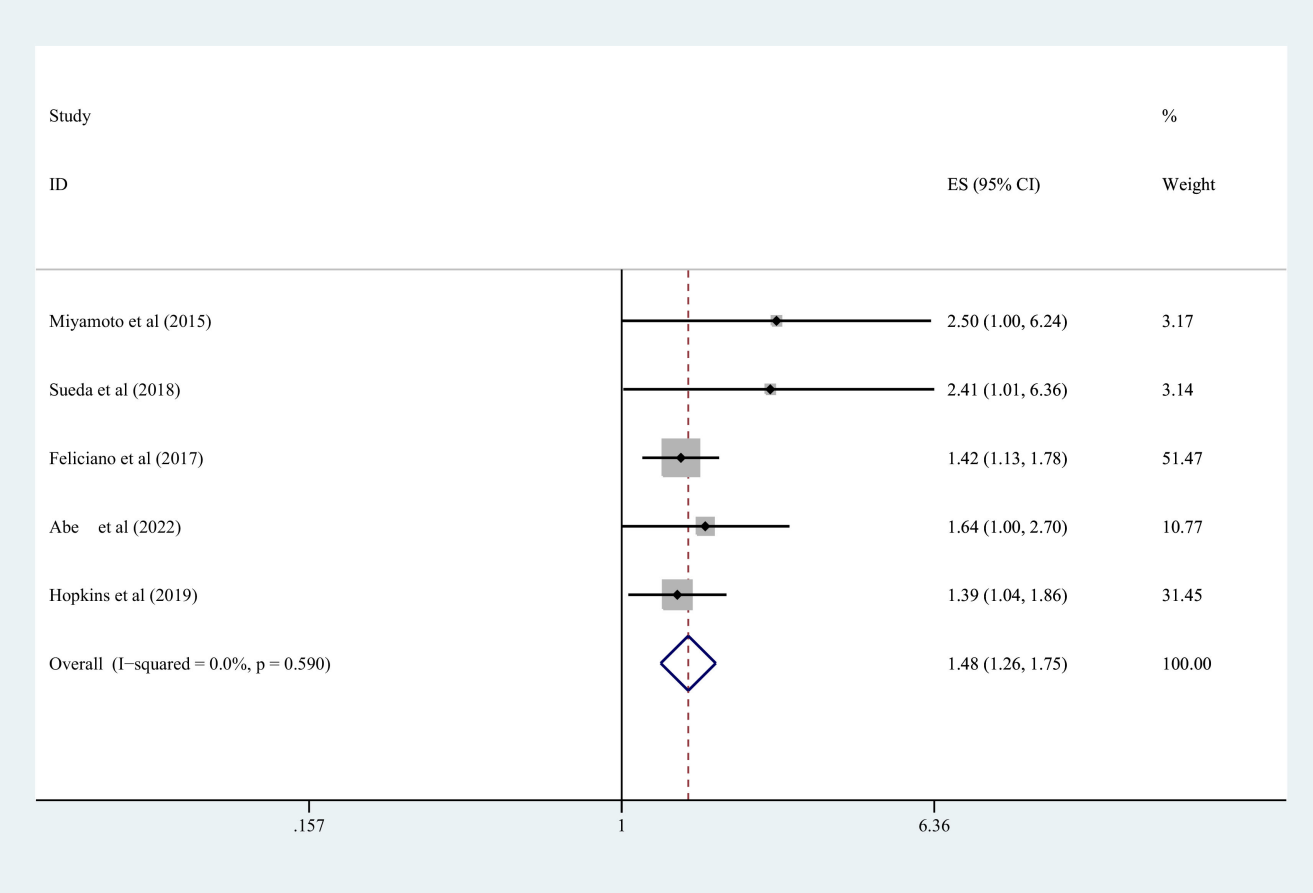
Forest plot of CSS risk in colorectal cancer patients with and without sarcopenia. CSS, cancer-specific survival.

Nineteen studies contributed to the survival outcome data. The meta-analysis depicted lowered OS in the sarcopenia group in comparison with the negative-sarcopenia group (HR=1.72,95% CI=1.45-2.03), (**Figure 3**). Additionally, 12 studies contributed data for multivariate analysis of DFS, and five studies contributed toward multivariate analysis of CSS. The meta-analysis depicted that compared with individuals without sarcopenia, individuals with sarcopenia had notably diminished DFS and CSS (DFS: HR=1.42,95% CI=1.26-1.60; CSS: HR=1.48,95% CI=1.26-1.75) (all *P*<0.001), as exhibited in **Figures 4**, **5**.

### 3.4 Subgroup analysis

A subgroup analysis of OS and DFS per tumor type, ethnicity, study type, the total number of included samples, NOS score, and diagnostic criteria for sarcopenia was performed. The data indicated an interesting trend in the sarcopenia group of lower OS and DFS in different subgroups. **Table 3** depicted the resulting data of the subgroup analysis in detail.

**Table 3:** Subgroup analyses in terms of overall survival and disease-free survival.

Characteristics	Overall survival				Disease-free survival			
	n	HR (95%CI)	P value	Heterogeneity	n	HR (95%CI)	P value	Heterogeneity
Study type								
Retrospective	17	1.73(1.43,2.08)	<0.001	63.1	11	1.41(1.25,1.50)	<0.001	50.8%
Prospective	2	1.75(1.32,2.30)	<0.001	0.0%	1	1.53(1.01,2.30)	0.04	NA
Patients number								
≥200	10	1.39(1.20,1.62)	<0.001	48.5%	6	1.33(1.17,1.51)	<0.001	6.6%
<200	9	2.57(1.92,3.45)	<0.001	19.2%	6	1.93(1.47,2.55)	<0.001	0.0%
NOS scores								
>5	14	1.71(1.39,2.10)	<0.001	57.9%	9	1.43(1.25,1.62)	<0.001	40.0%
≤5	5	1.91(1.30,2.79)	0.001	72.7%	3	1.39(1.07,1.82)	0.015	0.0%
Ethnicity								
Asian	12	1.78(1.37,2.31)	<0.001	66.2%	8	1.41(1.23,1.62)	<0.001	52.7%
Caucasian	7	1.68(1.33,2.12)	<0.001	53.7%	4	1.44(1.16,1.79)	0.001	0.0%
Criterion of sarcopenia								
=1	15	1.93(1.51,2.47)	<0.001	66.4%	9	1.40(1.23,1.60)	<0.001	20.5%
>1	4	1.41(1.21,1.63)	<0.001	14.2%	3	1.50(1.16,1.95)	0.002	74.7%
Tumor type								
Rectal cancer	11	2.37(1.61,3.48)	<0.001	70.8%	5	1.94(1.40,2.68)	<0.001	36.2%
Colorectal cancer	8	1.42(1.26,1.65)	<0.001	31.0%	7	1.36(1.20,2.42)	<0.001	0.0%

HR, hazard radio; NA, not applicable.

#### 3.4.1 According to tumor type

Nine studies explicitly included patients with rectal cancer, and the data indicated that individuals with rectal cancer in the sarcopenia group depicted shorter OS (HR=1.78, 95%CI=1.37-2.31, *P*<0.001) (**Figure 3**) and shorter DFS (HR=1.89, 95%CI=1.53-2.33, *P*=0.002) (**Figure 4**). Three studies did not explicitly differentiate into rectal and colon cancer, and the data indicated that individuals with colorectal cancer in the sarcopenia group depicted shorter OS (HR=1.42,95% CI=1.17-1.74, *P*<0.001) (**Figure 3**) and shorter DFS (HR=1.92,95% CI=1.52-2.42, *P*=0.001) (**Figure 4**).

#### 3.4.2 Analysis according to ethnic subgroups

The data depicted a shorter OS (HR=1.78,95%CI=1.37-2.31, *P*<0.001) in individuals with colorectal cancer in the sarcopenia group and a shorter DFS (HR=1.41,95%CI=1.23-1.62, P<0.001) in the Asian population. In the Caucasian population, individuals with colorectal cancer in the sarcopenia group also had shorter OS (HR=1.68, 95%CI=1.33-2.12, *P*<0.001) and shorter DFS (HR=1.44,95%CI=1.16-1.79, *P*=0.001) (**Table 3**).

#### 3.4.3 Subgroup analysis per the type of study

There were two main types of studies included in the literature, that is, prospective study and retrospective study. In the prospective study, individuals with colorectal cancer in the sarcopenia group had shorter OS (HR=1.75,95% CI=1.32-2.30, *P*<0.001) and shorter DFS (HR=1.53,95% CI=1.02-2.30, *P*=0.04). Whereas in the retrospective study, these patients depicted shorter OS (HR= 1.73,95%CI=1.43-2.08, P<0.001) and shorter DFS (HR=1.41,95%CI=1.25-1.59, P<0.001) (**Table 3**).

#### 3.4.4 Subgroup analysis according to the number of included samples

The data exhibited that individuals with colorectal cancer in the sarcopenia group had shorter OS (sample number >200: HR=1.39, 95%CI=1.20-1.62, *P*<0.001; sample number <200: HR=2.57, 95% CI=1.92-3.45, *P* < 0.001) and shorter DFS (sample number >200: HR=1.33, 95% CI=1.17-1.51, *P* < 0.001; sample number <200: HR=1.93,95% CI=1.47-2.55, *P* < 0.001) (**Table 3**).

#### 3.4.5 Subgroup analysis according to NOS score

In studies with NOS scores greater than 5, individuals with colorectal cancer in the sarcopenia group all had shorter OS (HR=1.71,95%CI=1.39-2.10, *P*<0.001) and shorter DFS (HR=1.43,95%CI=1.25-1.62, *P*<0.001). Additionally, in studies with NOS scores less than or equal to 5, sarcopenia colorectal cancer patients all had shorter OS (HR=1.91,95%CI=1.30-2.79, *P*=0.001) and shorter DFS (HR=1.39, 95%CI=1.07-1.82, P=0.015) (**Table 3**).

#### 3.4.6 The diagnostic criteria of sarcopenia

The resulting data depicted that sarcopenia colorectal cancer patients were linked with shorter OS (diagnostic criteria >1: HR=1.41,95% CI=1.21-1.63, *P*<0.001; diagnostic criteria=1: HR=1.93,95% CI=1.51-2.47, *P*<0.001) and shorter DFS regardless of which diagnostic criteria were followed (diagnostic criteria >1: HR =1.50,95% CI=1.16-1.95, *P*=0.002; diagnostic criteria=1: HR=1.40,95% CI=1.23-1.60, *P*<0.001) (**Table 3**).

### 3.5 Sensitivity analysis

**Figure 6:**
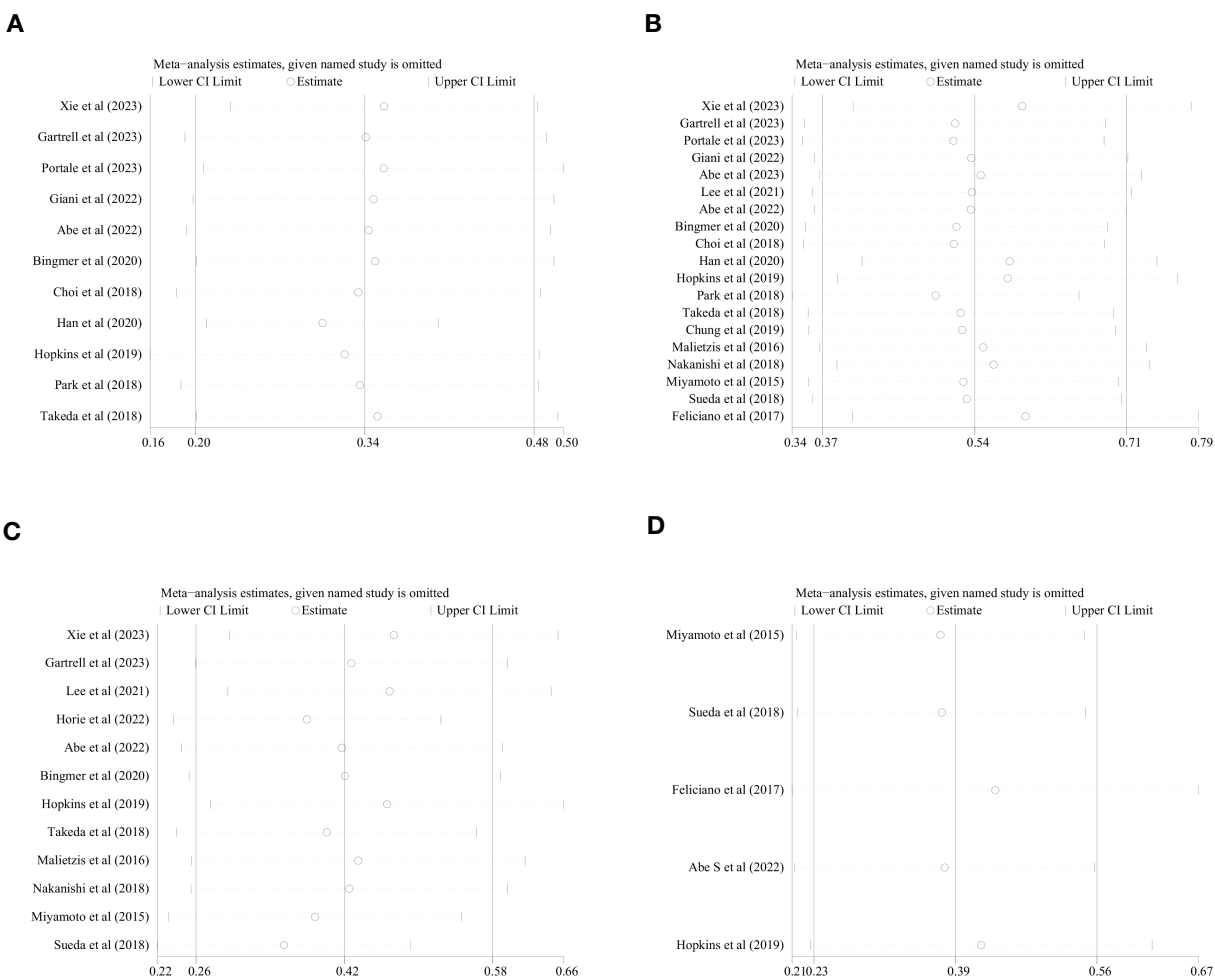
Sensitivity analysis of the meta-analysis. **(A)** incidence of sarcopenia, **(B)** OS, **(C)** DFS, **(D)** CSS.

The literature focusing on sarcopenia incidence, OS, DFS, and CCS was itemized and removed. Meta-analysis was executed on these remaining studies, and subsequent comparison of the resulting data between the remaining studies and the studies prior to exclusion was achieved. The comparison was indicative of the absence of any notable impact of itemized deletion of every study on the combined results. **Figures 6A–D** summarizes the sensitivity analysis of the meta-analysis for sarcopenia occurrence, OS, DFS, and CCS.

### 3.6 Publication bias

**Figure 7:**
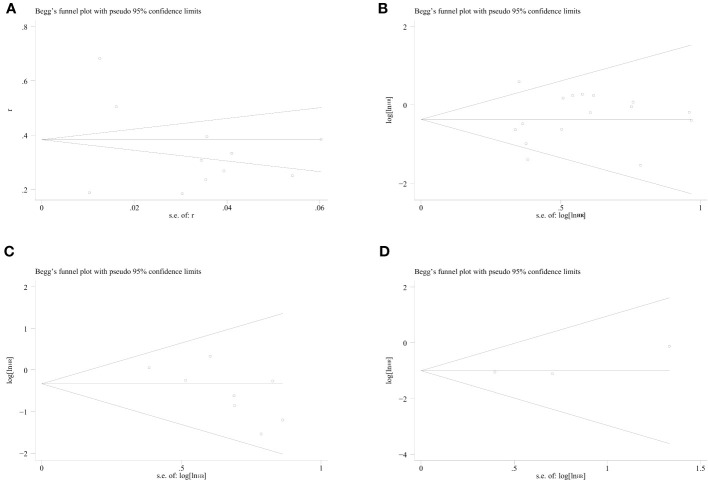
Funnel plots of sarcopenia incidence, OS, DFS, and CSS in colorectal cancer patients. **(A)** incidence of sarcopenia, **(B)** OS, **(C)** DFS, **(D)** CSS.

Meanwhile, no publication bias was noted concerning the incidence of sarcopenia per the respective Begg’s and Egger’s tests (*P*=0.76, *P*=0.74), OS (*P*=1.00, *P*=0.54), DFS (*P*=0.06, *P*=0.05) and CCS (*P*=0.30, *P*=0.42) (**Figures 7A–D**).

## 4 Discussion

This meta-analysis exhibited a relatively high incidence of sarcopenia in colorectal cancer (34%). In multivariate data, sarcopenia was linked with unfavorable OS, DFS, and CSS in individuals with colorectal cancer. This meta-analysis dealt with assessing the link between sarcopenia and OS, DFS, and CSS in individuals with colorectal cancer. The resulting data depicted congruence with the majority of prior observational studies. Hence, indicating a clinically meaningful relationship between sarcopenia and these survival parameters. The quality of evidence for the results of the multivariate analysis was considered reliable based on the NOS score.

In the past decade, research has increasingly demonstrated the predictive value of sarcopenia for the prognosis of cancer patients (39, 40). The occurrence of sarcopenia should not be neglected in clinical work-up, as the prevalence of sarcopenia in elderly individuals with cancer ranges from 12.5% to 57.7% (41). A meta-analysis depicted the presence of sarcopenia in 19%-39% of individuals with advanced colorectal cancer (42). Additionally, the proportion of colorectal cancer sarcopenia was as high as 34% in this study. This may be attributed to the fact that some of the patients included in the study received nCRT. Chemotherapy-related gastrointestinal reactions and radiotherapy-induced intestinal inflammation negatively affect the feeding habits and intestinal function of patients. These effects can result in reduced intake and impaired nutrient absorption, ultimately leading to weight loss and a high risk of malnutrition (43, 44). A study speculated upon the elements linked with sarcopenia prevalence in individuals with colorectal cancer. The resulting data of multivariate analysis determined that serum albumin, BMI, muscle wasting, and subjective overall patient scores were capable of independently predicting sarcopenia in individuals with colorectal cancer (45). Based on baseline data from our included studies (**Table 1**), individuals with sarcopenic colorectal cancer depicted a comparatively decreased BMI relative to non-sarcopenic patients. Therefore, variation in BMI could act as a clinical sign of early onset of sarcopenia in colorectal cancer patients. Diet and exercise are the two primary measures for preventing sarcopenia in individuals with cancer. Physical activity in such individuals is closely related to the maintenance of aerobic capacity and muscle strength (46). Hence, the proposal that appropriate nutritional supplementation and enhanced exercise can help improve the prognosis of colorectal cancer patients holds merit but needs to be verified in further clinical trials.

Cancer is the main factor contributing to the development of secondary sarcopenia. In current cancer research, sarcopenia diagnosis primarily relies on assessing decreased SMI, while fewer studies have evaluated muscle strength and physical performance (e.g., 4 m gait speed, 6 min walking test, and Time up and go test). To achieve more reliable conclusions, it is imperative to establish a more inclusive and comprehensive definition of sarcopenia. Since a CT scan is a routine method for the clinical staging of cancer patients, it is a practical technique for the assessment of body composition. The meta-analysis primarily explored the impact of sarcopenia on colorectal cancer prognosis, as a series of new studies have emerged in recent years and the conclusions may differ. In the 21 studies that were included, sarcopenia was measured by obtaining SMI values from CT scans of L3 levels, which is a common measure. The notion that it reduces heterogeneity and helps to draw relatively reliable conclusions may be plausible. These findings suggest that sarcopenia is negatively linked with the long-term prognosis of individuals with colorectal cancer. Additionally, research has exhibited that sarcopenia in individuals with cancer is linked with an elevated risk of cardiovascular disorders (47). Additionally, Nipp et al. (48) noted that sarcopenia influenced depression and quality of life in individuals with advanced cancer. Thus, the detrimental impact of sarcopenia highlights the significance of preventive measures and therapeutic interventions for sarcopenia among healthcare providers and individuals with cancer. To date, the reason for the negative association of sarcopenia with prognosis in individuals with colorectal cancer remains unclear. Abe S et al. (30) suggested that overall and grade 3 or higher variables of Common Terminology Criteria for Adverse Events were not increased in the post-chemoradiotherapy sarcopenic patients. However, Nakanishi et al. (37) thought that sarcopenia was significantly correlated with infections at locations other than surgical sites. Sun et al. observed that patients with sarcopenia showed a significant higher rate of incidence of postoperative infected when compared with non-sarcopenia patients by means of meta-analysis (17). A post-operative infection can affect long term survival. This may partly explain why patients with sarcopenia have worse prognosis. On the other hand, it can be speculated that systemic inflammation could possibly be one of the causes. The systemic inflammatory response severely affects muscle protein degradation metabolic processes and accelerates muscle wasting (49). Sarcopenia is indicative of an elevated catabolic state and is linked to a heightened inflammatory response following colorectal cancer surgery (50, 51). Moreover, the presence of both inflammation and sarcopenia contributes to a heightened risk of mortality (52). However, the precise mechanism needs further study.

This meta-analysis is limited in some aspects. Firstly, all the assessed studies used cut-off values of SMI to define sarcopenia. However, many studies did not address the assessment of muscle strength and fitness and should be supplemented with diagnostic criteria for sarcopenia according to EWGSOP. Second, the cut-off value of SMI was diverse across studies, which may be a source of heterogeneity. Moreover, neoadjuvant radiotherapy and nCRT are also vital elements affecting the prognosis of individuals with colorectal cancer. Nonetheless, the absence of comprehensive information and data based on neoadjuvant or adjuvant chemotherapy was noted in the studies assessed. Therefore, there is a possibility of these variables influencing the resulting data. Thus, future studies incorporating individual-level data are essential to validate our findings. Ultimately, we cannot perform a subgroup analysis based on the primary site of tumor occurrence, for example, rectal cancer, right hemicolectomy, or left hemicolectomy. Patients with colorectal cancer have different prognoses depending on the primary site. Though we designated the adjusted HR value as effect value, distant metastasis, lymph node metastases, involved surgical margin, lymph vascular invasion and histological grade were still dominant factors affecting survival time. The effect of sarcopenia on colorectal cancer should be framed in the context of former factors. Therefore, confounding baseline information may limit our ability to further explore the relevance of sarcopenia in colorectal cancer.

## 5 Conclusion

Sarcopenia is more prevalent in individuals with colorectal cancer. In such individuals, sarcopenia is independently linked with an unfavorable prognosis. Future studies need a comprehensive definition of sarcopenia to strengthen the evidence and further validate our conclusions.

## Data availability statement

The original contributions presented in the study are included in the article/supplementary material. Further inquiries can be directed to the corresponding author.

## Author contributions

JH developed the research idea, performed data collection, performed data analysis, prepared the first manuscript draft, refined the research idea, and edit manuscript. WL, YH, YM, and LS validated data collection, developed the research idea, and proofread the article. All authors contributed to the article and approved the submitted version.
